# Mechanisms and novel therapeutic roles of bitter taste receptors in diseases

**DOI:** 10.7150/thno.107406

**Published:** 2025-03-03

**Authors:** Aiyang Tong, Hongyu Yang, Xiaohui Yu, Dongkai Wang, Jian Guan, Ming Zhao, Ji Li

**Affiliations:** Department of Pharmaceutics, School of Pharmacy, Shenyang Pharmaceutical University, No. 103, Wenhua Road, Shenyang, 110016, P. R. China

**Keywords:** TAS2R, Signaling mechanism, Extraoral expression, Disease management, Drug delivery

## Abstract

Bitter taste receptors (TAS2R) are expressed in the oral cavity, intestine, airways, and vascular smooth muscle, where they regulate physiological processes, including immune responses. However, the activation of TAS2R triggers signaling pathways that influence inflammation, metabolism, and cell proliferation, suggesting their potential as therapeutic targets for diseases such as Alzheimer's disease, Parkinson's disease, asthma, and cancer. Bitter compounds capable of activating TAS2R have shown potential in modulating these pathways, presenting novel opportunities for drug development. This review examines the expression of TAS2R across diverse tissues, their complex physiological roles, and potential therapeutic applications, including disease management and targeted drug delivery.

## 1. Introduction

**Figure 1:**
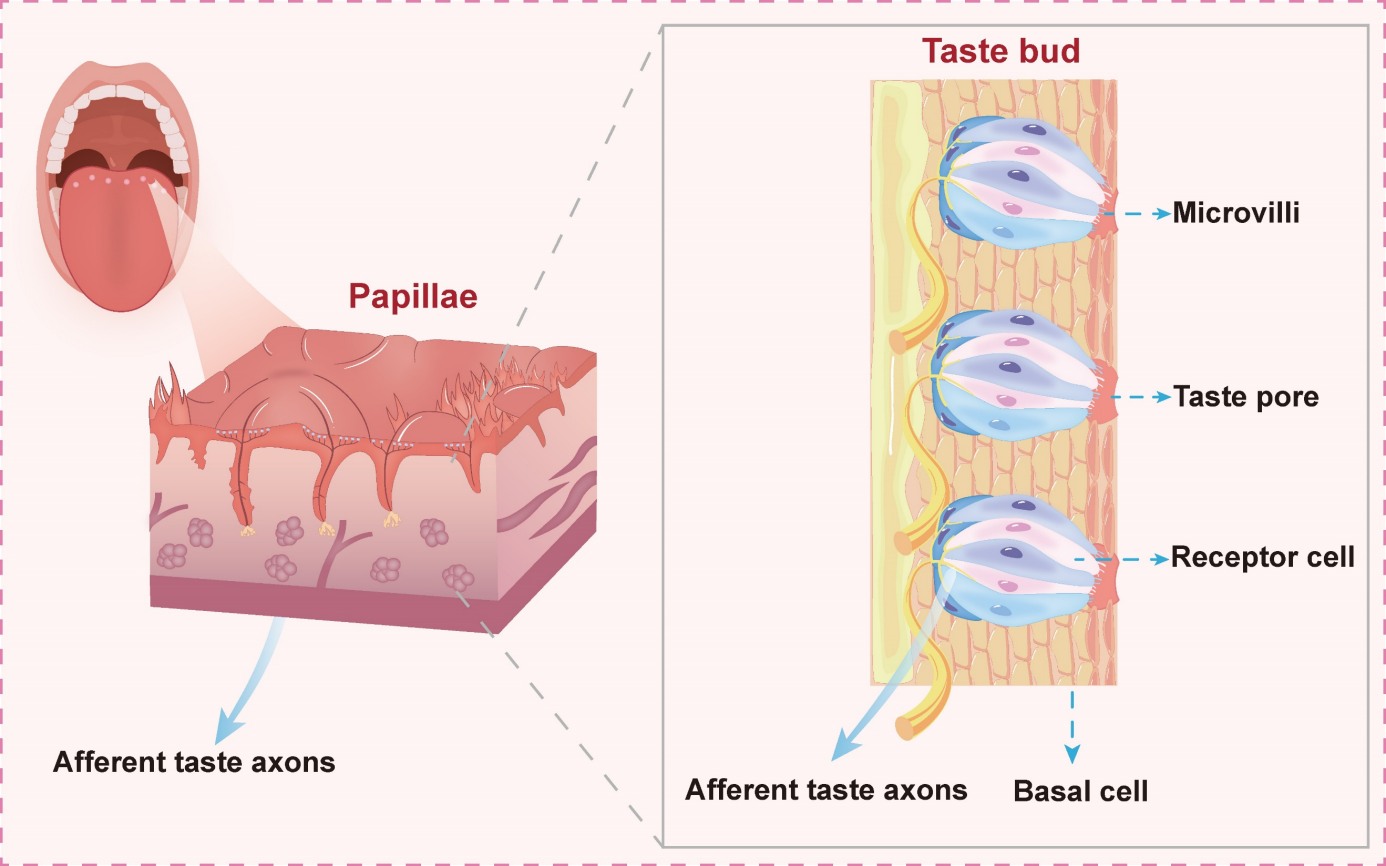
**Schematic illustration of taste bud organization on the tongue.** Lingual papillae housing taste buds are shown with structural components including microvilli, taste pores, and receptor cells, which mediate signal transduction through gustatory afferent nerve fibers to central processing centers.

Taste receptors, including ion channels and G protein-coupled receptors (GPCRs), detect sour, bitter, salty, sweet, and umami flavors. It was initially identified as a taste receptor expressed in small populations of specialized epithelial cells on the tongue (**Figure 1**), these receptors are now recognized for their broader roles beyond oral taste perception. Their detection functions extend throughout the digestive tract, including the intestinal epithelium, respiratory tract, and gums, where they sense various metabolites involved in interactions between mucosal surfaces and microorganisms 1. For instance, isolated chemosensory cells (SCCs) and tuft cells expressing taste-signaling proteins are present in several mucous membranes. SCCs can detect allergens, bacteria, noxious stimuli, viruses, driving avoidance behavior, antimicrobial responses, and neuroinflammation in the airway. Similarly, tuft cells in the intestine sense helminth infections and bacterial dysregulation, triggering a type II immune response characterized by tissue remodeling. In the gums, SCCs detect disease, evoke innate immune responses, and release antimicrobial compounds in the epithelium, thereby regulating the composition of the microbiome 2.

Taste cells can be categorized into three distinct types based on morphology and function: Type I, Type II, and Type III. These specialized cells are responsible for detecting the five basic tastes. The sensation of "bitter" is mediated by specific GPCRs expressed in certain Type II cells 3. TAS2R, which are GPCRs, are encoded by approximately 27 to 51 genes. These receptors are localized on the surface of taste buds and exhibit a characteristic structure comprising seven transmembrane helices formed by a single polypeptide chain 4, 5. To date, 25 TAS2R genes have been identified in humans, with their genomic locations mapped to chromosomes 5p15, 7q31, and 12p13. Some TAS2R, such as TAS2R10, TAS2R14, and TAS2R46, can recognize multiple compounds, while others demonstrate strict specificity for a single bitter compound 6.

TAS2R is expressed in various tissues and cells throughout the human body, including the gastrointestinal tract, skin, tuft cells and tumor cells. The activation of TAS2R in tuft cells can elicit a type 2 immune response effective in treating parasitic infections 7. Additionally, TAS2R may aid in managing obesity by repairing intestinal barrier integrity, upregulating GLP-1 release, and promoting tuft cell proliferation 8. In keratinocytes, TAS2R function as intracellular “gatekeepers” facilitating the excretion of harmful substances via ABCB1, enhancing our understanding of their role in non-gustatory tissues and laying the groundwork for novel drugs to bolster the skin's defense against harmful substances 9. Furthermore, TAS2R's involvement in regulating tumor cell apoptosis signifies their significant research value 10, 11. In this review, we provide a detailed overview of the signaling mechanisms of TAS2R and explore their roles in tissues and cells outside the oral cavity. TAS2R are expressed in a wide array of tissues, and this broad tissue distribution suggests that TAS2R could play a crucial role in modulating various physiological processes. Consequently, TAS2R represents a promising target for the development of novel therapeutic strategies.

## 2. Signaling mechanism of TAS2R

### 2.1 TAS2R-PDE-cAMP pathways

TAS2R activation follows dual signaling mechanisms through G-protein-coupled pathways, inducing rapid second messenger changes. In one pathway, bitter compounds such as cycloheximide bind to TAS2R, activating the G protein's α-subunit. This activation reduces intracellular cAMP levels via phosphodiesterase stimulation 12. Consequently, cAMP-dependent ion channels are inactivated, triggering the release of intracellular calcium stores, increasing cytosolic Ca²⁺ concentration, and causing membrane depolarization.

### 2.2 TAS2R-PLCβ2-IP3 Q pathways

Another mechanism involves the binding of bitter compounds to TAS2R, which activates the β-γ subunit of the gustatory G protein. This activation induces the synthesis of phospholipase Cβ2 (PLCβ2) through inositol 1,4,5-triphosphate (IP3), leading to the release of intracellular Ca²⁺. The increased Ca²⁺ levels depolarize bitter taste receptor cells, prompting neurotransmitter release 13. Interestingly, studies on Gustducin-deficient mice suggest alternative pathways, where specific bitter substances directly stimulate TAS2R, bypassing Gustducin and activating ion channels 14.

### 2.3 The role of multiple signal transduction pathways in TAS2R

**Figure 2:**
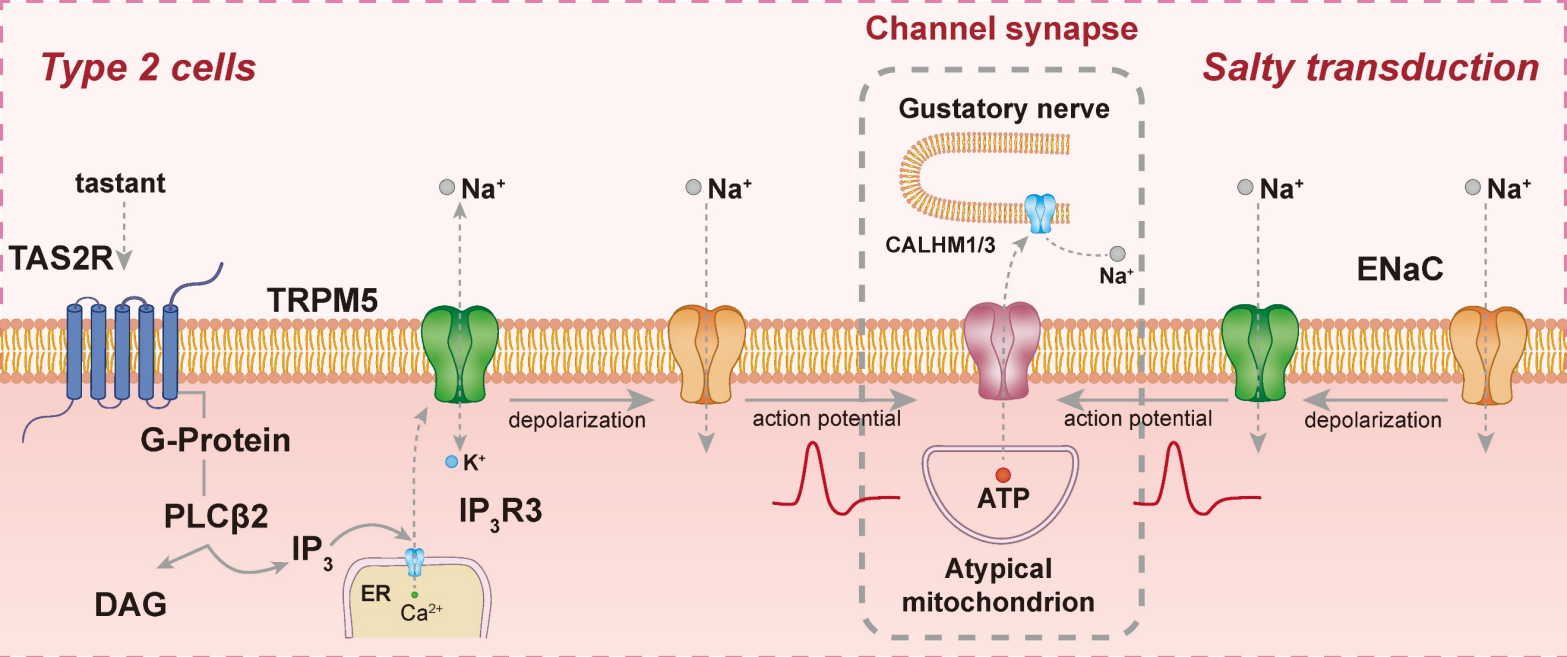
**Signal transduction pathways of TAS2R.** Bitter substances bind to TAS2R, activating PLCβ2 to produce IP3 and DAG, leading to Ca²⁺ release and TRPM5 activation, which causes depolarization and action potential generation. Salty stimuli modulate ENaC channel activity, directly depolarizing taste cells to trigger action potentials. Synaptic transmission is mediated by CALHM1/3 channels, facilitating neurotransmitter release to gustatory afferent neurons.

Certain substances, such as quinine, can close K⁺ channels, resulting in the depolarization of TAS2R-expressing cells 15. Additionally, bitter compounds can activate G proteins and PLCβ2 by binding to TAS2R, thereby elevating intracellular Ca²⁺ concentrations and leading to the activation of TRPM4 and TRPM5, resulting in Na⁺ influx 13. These findings highlight the existence of multiple transduction pathways for a single substance, revealing interdependent mechanisms and shared signaling components (**Figure 2**).

## 3. Extra-oral role of TAS2R

Initially, TAS2R was believed to help animals avoid toxic compounds such as strychnine and nicotine 16. When a bitter compound binds to TAS2R, it activates the receptors, initiating a sensory response. The chords tympani and glossopharyngeal nerves transmit this signal to the brain for processing 3. However, not all toxic compounds taste bitter, and a bitter taste does not always signify toxicity 4. Further research has revealed that TAS2R is expressed beyond the oral cavity, including in the intestine, airways, urinary tract, vascular and respiratory smooth muscle, nervous system, and thyroid gland. TAS2R at these diverse sites performs distinct regulatory roles in various physiological processes (**Table 1**).

### 3.1 Role of TAS2R in neurodegenerative diseases

Brain disorders pose a significant global healthcare challenge, with drug delivery across the blood-brain barrier remaining a critical obstacle. Characteristic features of central nervous system (CNS) disorders include protein misfolding and cell death, often aggravated by neuroinflammation and oxidative stress 66. Therein, Alzheimer's disease (AD) and Parkinson's disease (PD) are two of the most common neurodegenerative diseases worldwide, typically characterized by progressive neuronal loss and functional decline.

#### 3.1.1 Role of TAS2R in Alzheimer's disease

**Figure 3:**
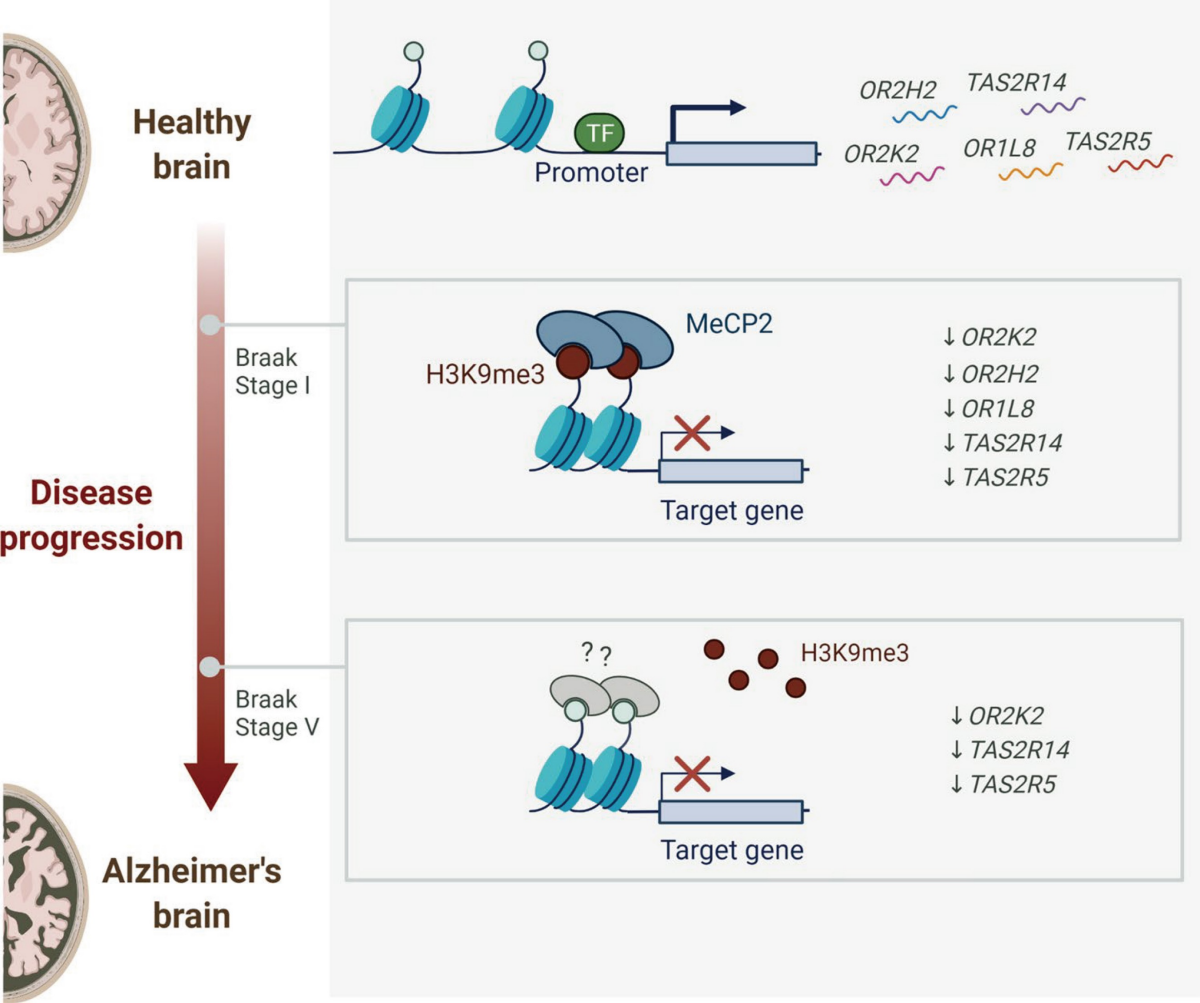
**Dynamic alterations in receptor expression during Alzheimer's disease progression.** Healthy brains constitutively express olfactory receptors (OR2H2, OR1L8) and bitter taste receptors (TAS2R14, TAS2R5). Early Braak stages (I-V) exhibit progressive downregulation of OR2K2, OR2H2, OR1L8, TAS2R14, and TAS2R5, with persistent OR2K2 suppression observed in advanced neuropathological stages. Adapted with permission from 71, copyright 2023, Springer.

AD, one of the most widespread neurodegenerative disorders, progresses to Alzheimer's dementia, characterized by the progressive impairment of cognition and functional capacity, ultimately compromising patient independence 67. Increasing evidence suggests that genetic, cellular, and circuitry dysregulation contribute to both the cellular pathology and cognitive deficits observed in AD 68. Neuroinflammation plays a pivotal role in AD pathogenesis, with microglia, the resident innate immune cells of the CNS, serving a key role in this process. Extracellular β-amyloid plaques interact with receptors for advanced glycation end products (RAGE) on microglia, leading to their overactivation and the subsequent release of pro-inflammatory factors. This heightened inflammatory state can alter brain energy metabolism, impair neuronal network function, and compromise the integrity of the blood-brain barrier 69. Pathophysiological changes involving alterations in specific proteins lead to dysfunction across various cellular pathways, including mitochondrial dysfunction, excitotoxicity, synaptic dysfunction, damage to protein degradation systems, endoplasmic reticulum (ER) stress, DNA damage, inflammation and increased levels of reactive oxygen species (ROS) due to cell cycle re-entry 70. These changes contribute to the development of AD. Furthermore, Alves *et al.* 71 reported a correlation between TAS2R expression modulation and AD progression, as shown in **Figure 3**. TAS2R genes, along with other AD-associated genes, were observed to be progressively downregulated from the early stages of AD, with continued decline as the disease advanced. This indicates that TAS2R may serve as both a potential biomarker and a therapeutic target, warranting further investigation. However, the precise role of TAS2R in AD pathophysiology remains poorly understood, emphasizing the need for further studies to elucidate the mechanisms linking its expression to disease progression.

#### 3.1.2 Role of TAS2R in Parkinson's disease

PD is the second most prevalent neurodegenerative disorder after AD, presenting as a distinct clinical syndrome 72. As in AD, a key molecular pathway in PD involves α-synuclein aggregation, which triggers NLRP3 inflammasome activation. The fibrillation of α-synuclein has been shown to induce a delayed yet robust activation of the NLRP3 inflammasome in primary mouse microglia 73.

Alterations in TAS2R expression have been observed in PD patients 74, 75, with downstream signaling pathways involved in inflammatory responses suggesting potential therapeutic applications. NF-κB and the NLRP3 inflammasome are central regulators of inflammation in human cells, contributing to both neuroinflammation and TAS2R signaling dysregulation. NF-κB and the NLRP3 inflammasome are central regulators of inflammation in human cells, contributing to both neuroinflammation and TAS2R signaling dysregulation. NF-κB, a key regulator of inflammatory transcription networks, controls the expression of genes involved in immune responses, inflammation, and oxidative stress following exposure to pathogens, cytokines, and reactive species. IL-1β, a pro-inflammatory cytokine, may be produced due to TAS2R signaling defects and can activate NF-κB via Toll-like receptor pathways 76. In a 2011 study, Nisha Singh *et al.* 77 discovered TAS2R expression in various regions of the rat brain. RT-PCR analysis confirmed the expression of TAS2R4, TAS2R48, and TAS2R107 in the brainstem, cerebellum, cortex, and ventral nucleus. TAS2R activation has been linked to memory improvement by reducing IL-1β and TNF-α levels and mitigating LPS-induced neuroinflammation, oxidative stress, and apoptosis 78.

**Figure 4:**
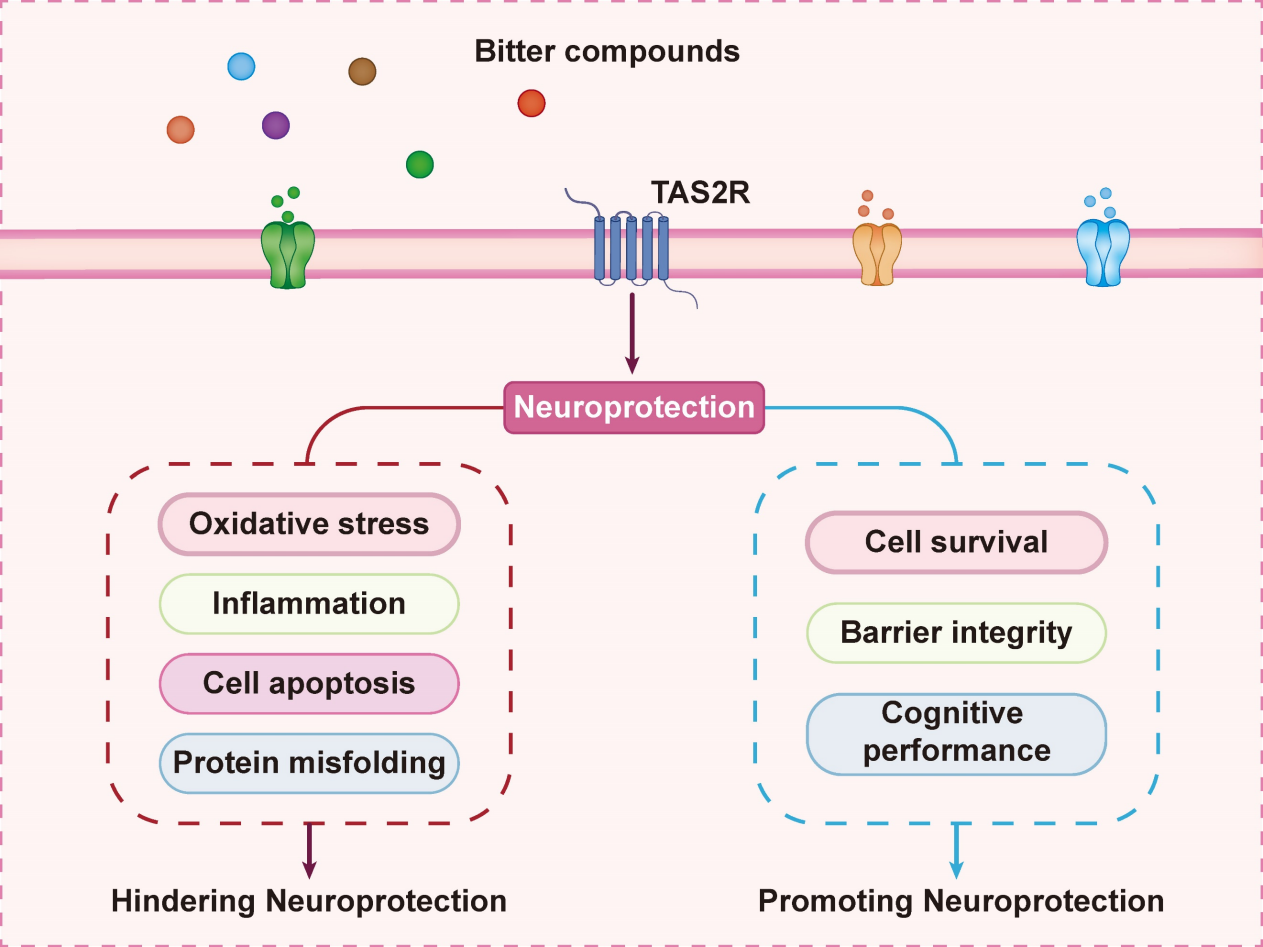
** Multifaceted neuroprotection mediated by TAS2R receptors.** TAS2R signaling orchestrates neuronal protection through dual mechanisms: (1) suppressing oxidative stress, neuroinflammation, apoptotic signaling, and protein misfolding pathologies; (2) enhancing cell survival pathways and integrity while improving cognitive function.

Certain TAS2R agonists have been shown to suppress NLRP3 and NF-κB signaling, suggesting their potential as therapeutic agents to mitigate neuroinflammation in CNS disorders. Accordingly, bitter receptor agonists have been demonstrated to partially downregulate inflammatory pathways by inhibiting NLRP3, NF-κB, and related oxidative stress markers (**Figure 4**). Efforts are underway to identify new therapeutic strategies for neuroinflammatory diseases using cannabinoids, phenols, and endogenous anti-inflammatory cytokines 79. TAS2R regulates multiple biological processes initiated by bitter taste ligands in the brain, and bitter compounds are emerging as promising candidates for CNS disease treatment. Further research is required to elucidate the transport mechanisms of bitter compounds across the blood-brain barrier, as their limited bioavailability remains a challenge.

### 3.2 The role of TAS2R in chronic otitis media

Chronic otitis media (COM), a persistent ear, nose, and throat disorder, significantly affects individuals' health. It remains prevalent both domestically and internationally, being a primary cause of hearing loss 80. Key aspects of its pathogenesis involve mucosal hyperplasia and leukocyte infiltration in the middle ear, conditions that typically resolve with bacterial clearance through apoptosis. Activating innate immune receptors during the inflammatory phase leads to the recruitment of intracellular transcription factors (e.g. NF-κB, AP-1), which regulate the inflammatory response and influence the proliferation of tissue cell lineages 81.

To assess the presence of TAS2R in the middle ear and its potential link with COM, Kaufman *et al.* 82 recruited 84 volunteers, detecting TAS2R expression in all participants' middle ear samples. The rs1376251 allele of TAS2R50 was identified as being associated with chronic otitis media. However, while the study identified an association, further investigation is required to determine causality and clarify the mechanisms through which TAS2R50 and its alleles contribute to COM pathophysiology. A deeper understanding of these mechanisms could support the development of TAS2R-targeted therapies aimed at modulating inflammatory responses and improving outcomes for patients with COM.

### 3.3 Role of TAS2R in nasal inflammation

Chronic rhinosinusitis (CRS) is a complex upper respiratory disease characterized by persistent bacterial or fungal infections due to impaired mucociliary clearance 83. Traditionally, primary CRS has been classified into two main categories: CRS with nasal polyps and CRS without nasal polyps, with eosinophilic sinusitis and allergic fungal sinusitis identified as additional subtypes 84. Allergic rhinitis (AR) is an immunoglobulin E (IgE)-mediated inflammatory condition triggered by allergen exposure. Upon contact with an allergen, the immune system generates IgE, which binds to mast cells and basophils. Upon re-exposure, allergen binding to IgE triggers a Type I hypersensitivity reaction, leading to histamine release and the activation of immune cells and cytokines involved in nasal mucosal inflammation 85. Common symptoms include sneezing, nasal congestion, itching, and a runny nose, affecting over 400 million people worldwide 86. Inflammatory nasal diseases like CRS and AR lack a definitive cure and significantly impact patients' quality of life, increase healthcare costs, and reduce productivity 87. In recent years, research has highlighted the role of taste receptor signaling in modulating mucosal immunity, positioning this pathway as a promising therapeutic target for respiratory diseases. Early studies identified TRPM5, a transient receptor potential ion channel, as crucial for bitter taste perception and highly expressed in the mouse nasal cavity 88. Expanding on this, Dr. Henry P. Barham discovered that TAS2R46, TAS2R14, and TAS2R4, along with downstream components such as α-Gustducin, PLCβ2, and TRPM5, are expressed in human nasal structures, including the inferior and middle turbinate and the nasal septum 89. Cilia, the first line of defense against inhaled pathogens, play a critical role in airway protection. TAS2R are expressed on active airway cilia, where they initiate innate immune responses by enhancing ciliary beating frequency (CBF) through guanylyl cyclase and protein kinase G activation, ultimately improving mucosal clearance 90. The TAS2R14 agonist DPD enhances both CBF and nitric oxide production at the air-liquid interface (ALI) in the nasal cavity, inhibiting bacterial growth and biofilm formation 91.

Recently, studies by Freund *et al.* and Hariri *et al.* further demonstrated the expression of TAS2R4, TAS2R14, and TAS2R16 in sinus cilia, with activation by quinolone derivatives such as 2-heptyl-3-hydroxy-4-quinolone and 4-hydroxy-2-heptylquinolone 92, 93. Notably, certain TAS2R, such as TAS2R39, are upregulated in response to inflammatory cytokines, including IL-3, IL-5, IL-10, and TGF-β. Bitter compounds can induce nasal mucosal constriction, with similar effects observed in rat models. However, the upregulation of several TAS2R during inflammation suggests a dual role, contributing to both nasal mucosal contraction and the pathogenesis of inflammatory diseases 94, 95. TAS2R plays a crucial role in CRS and AR, as their activation enhances ciliary activity, aiding pathogen clearance. However, the involvement of TAS2R in inflammatory cytokine regulation warrants further investigation to clarify their complex roles and therapeutic potential in inflammatory nasal diseases.

### 3.4 Role of TAS2R in asthma

**Figure 5:**
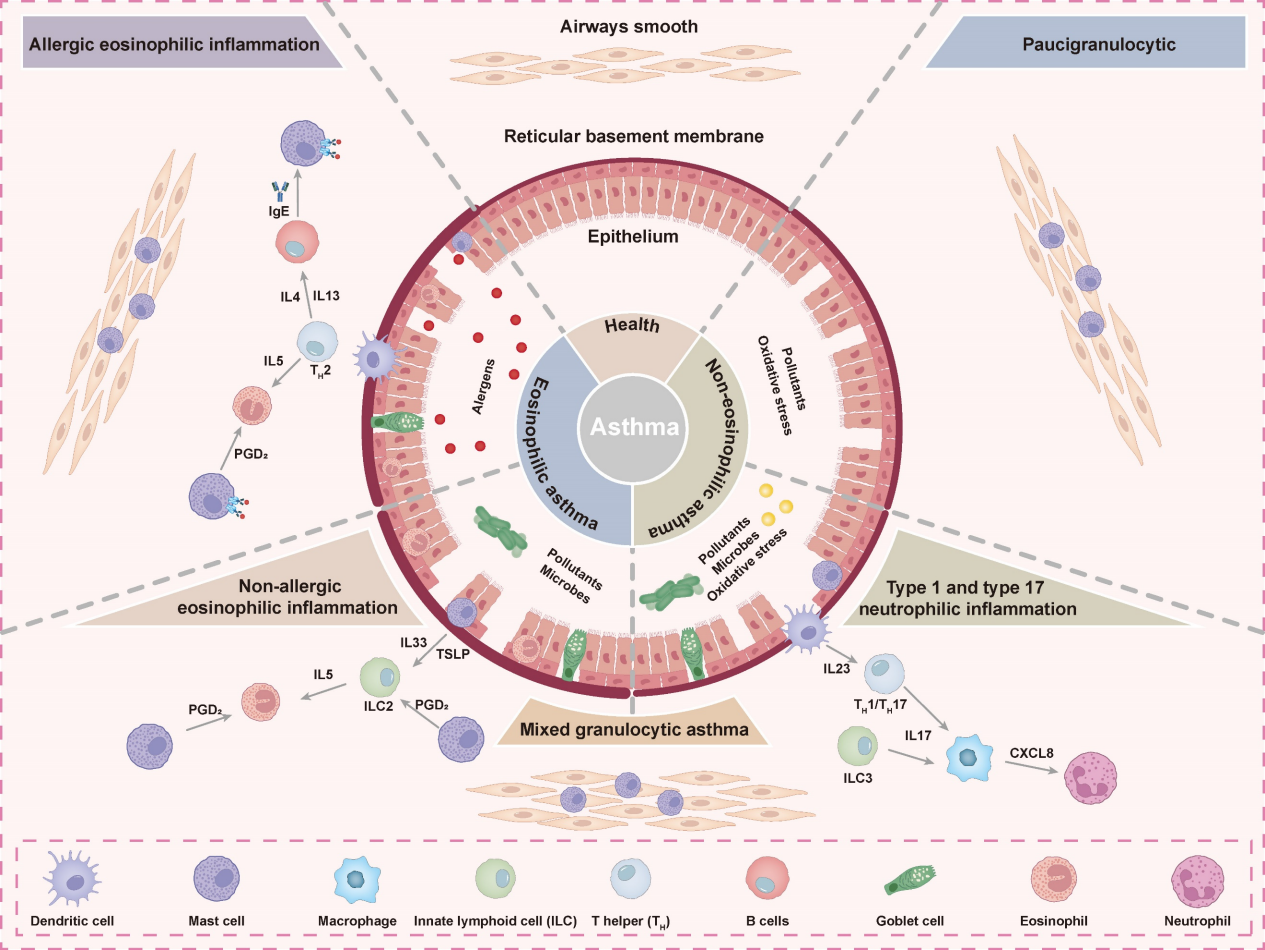
** Mechanisms and characteristic pathological features of the immunopathology of asthma.** Asthma includes allergic eosinophilic, non-allergic eosinophilic, mixed granulocytic, and type 1 and type 17 neutrophilic phenotypes. Each phenotype is linked to specific cell types and mediators. Asthmatic airways show changes in epithelial cells, basement membrane, and smooth muscle compared to healthy airways.

Asthma is a chronic respiratory syndrome characterized by airway inflammation, nonspecific airway hyperresponsiveness, and reversible airway obstruction, with symptoms including wheezing and shortness of breath 96, 97. Bronchial asthma involves multiple inflammatory factors and immune cells and can be classified into paucigranulocytic asthma (PGA), eosinophilic asthma (EA), mixed granulocytic asthma (MGA), and neutrophilic asthma (NA) based on distinct inflammatory profiles in induced sputum **(Figure 5)** 98. Among these, eosinophils are key inflammatory cells and can be further categorized into allergic and non-allergic subtypes 99. Asthma results from complex interactions between genetic and environmental factors. Exacerbations often involve airway remodeling, characterized by airway smooth muscle (ASM) thickening, excessive mucus secretion, and mucosal angiogenesis, which collectively contribute to airway stiffening and irreversible airway obstruction 100. Current treatments, such as β2-agonists and corticosteroids, focus on bronchoconstriction relief but have limited efficacy against airway remodeling due to β2-agonist a number of adverse effects 23. Therefore, the identification of novel therapeutic targets is crucial for addressing airway remodeling in asthma. Recent studies have identified TAS2R as promising therapeutic targets because of their expression on ASM cells and their capacity to induce ASM relaxation and airway dilation upon activation 101.

**Figure 6:**
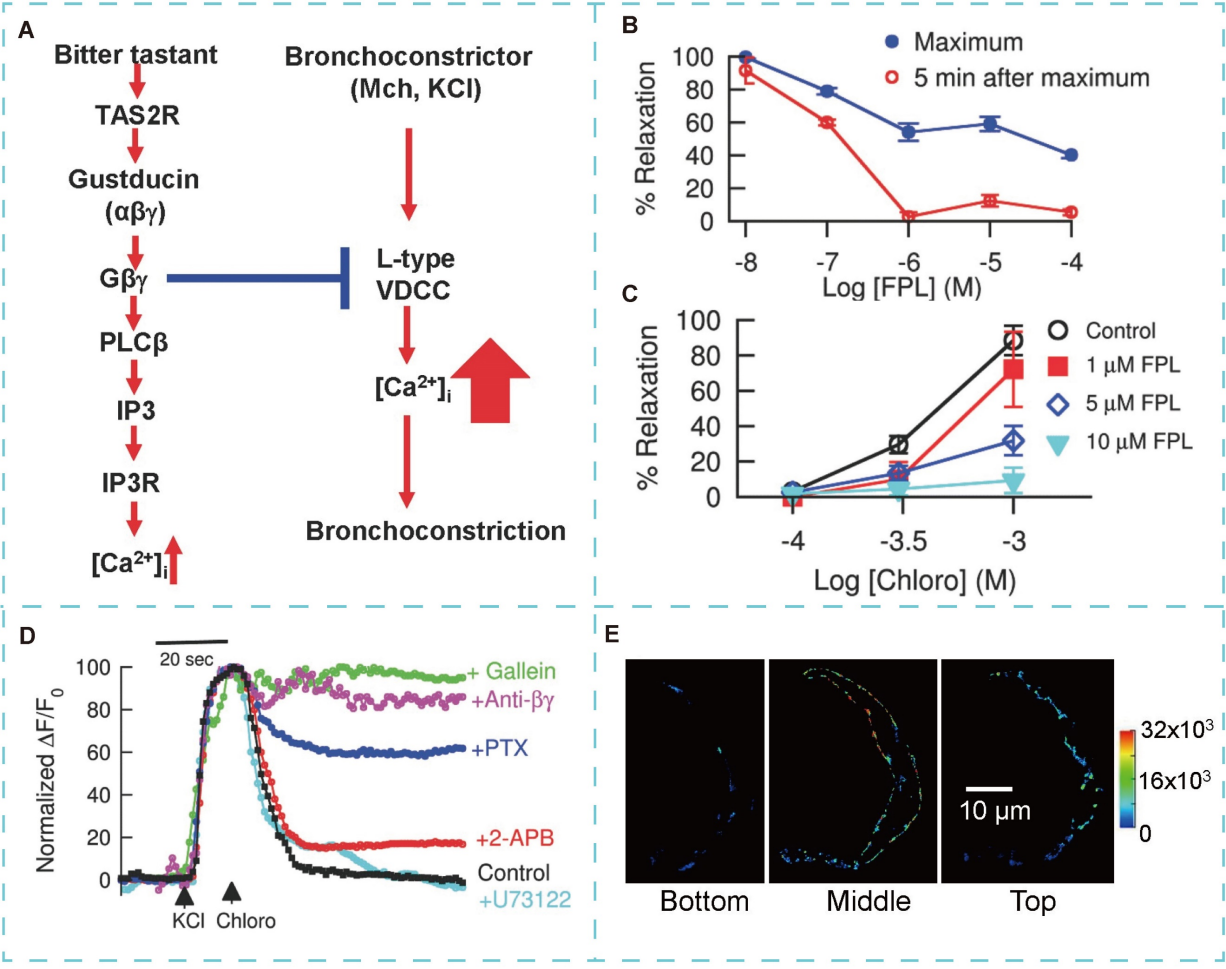
**The basis of TAS2R induced bronchodilation. (A)** A model for TAS2R signaling and bitter tastant-induced bronchodilation. **(B)** FPL 64176 (FPL) dose-dependently reversed chloro-induced bronchodilation in Mch precontracted airways. **(C)** FPL dose-dependently inhibited chloro-induced bronchodilation of KCl-precontracted airways. **(D)** Representative recordings of changes in [Ca^2+^]. **(E)** Cellular distribution of TAS2R107 in three planes of an isolated mouse ASM cell. Adapted with permission from 104, copyright 2013, Public Library of Science.

Deshpande *et al.* 102 demonstrated that TAS2R is expressed in the ASM of the human lung. The expression of TAS2R in ASM renders them potential drug targets for asthma treatment. Studies have shown that bitter taste receptor agonists induce relaxation and dilation of isolated ASM, with effects three times more pronounced than those induced by beta-agonists. Recently, TAS2R agonists have emerged as potential direct bronchodilators, among the 25 TAS2R isoforms, three—TAS2R10, TAS2R14, and TAS2R31—are notably overexpressed in ASM. Unlike β2 receptors, TAS2R-induced airway dilation occurs independently of cAMP increase. This suggests that the mechanism of TAS2R-induced airway relaxation is not mediated through an elevation in cAMP levels 103. Upon TAS2R activation by bitter substances, the associated G-protein subunit Gαt dissociates from the TAS2R-Gαt/Gβγ complex. The released Gαt binds to the acetylcholine receptor (AchR), competitively inhibiting the function of the Gq protein coupled to AchR. This prevents calcium signaling initiated by AchR activation, resulting in ASM relaxation 101. TAS2R agonism enhances cofilin activation, a key regulatory protein of the actin cytoskeleton, leading to ASM relaxation 106. Additionally, TAS2R activation increases local Ca^2+^ concentration, triggering ASM relaxation by activating large-conductance Ca^2+^-activated K^+^ channels (BKCa) 102. Furthermore, Zhang *et al.* 104 demonstrated that bitter substances induce bronchodilation by reversing the calcium concentration increase caused by bronchoconstrictors (**Figure 6**).

A key feature of airway remodeling in asthma is the proliferation of ASM, manifested as both hypertrophy and hyperplasia. A significant number of patients with severe asthma exhibit ASM proliferation. The drugs currently used to treat asthma range from beta-agonists to corticosteroids, with protein kinase A (PKA) playing a pivotal role in mediating the effects of beta-agonists on ASM function. However, PKA activity induced by beta-agonists in ASM cells appears to be desensitized to beta2-adrenoceptors, rendering beta-agonist-based antimitogenic drugs relatively weak and with limited impact on remodeling. Pawan Sharma *et al.* 105 utilized a TAS2R agonist to pre-treat airway smooth muscle (ASM) cells isolated from both healthy controls and asthmatic patients. This treatment demonstrated a dose-dependent suppression of ASM proliferation, indicating a significant anti-mitogenic effect of TAS2R agonists. Specifically, TAS2R agonists were found to block the downstream signaling of phosphatidylinositol 3-kinase and reduce the phosphorylation of growth factor-stimulated protein kinase B (Akt), without inhibiting the supply of phosphatidylinositol 3,4,5-trisphosphate. Collectively, TAS2R agonists suppress ASM proliferation in a dose-dependent manner, demonstrating significant anti-mitogenic effects. This suggests the existence of new receptor targets and signaling pathways that could mitigate airway remodeling and bronchoconstriction in asthma. These findings highlight TAS2R as a promising therapeutic target for asthma, potentially providing an alternative to current treatments.

### 3.5 The role of TAS2R in metabolic diseases

#### 3.5.1 Diabetes mellitus type 2

The aetiopathology of type 2 diabetes mellitus (T2DM) is characterized by insulin resistance, initially coupled with hyperinsulinemia, followed by a progressive decline in the insulin-producing capacity of pancreatic beta cells. In addition, dysregulation of incretin hormones, lipolysis, hyperinsulinemia, enhanced renal glucose reabsorption, and disrupted central appetite regulation are key factors in the pathophysiology of T2DM 106. Incretin hormones, secreted following food intake, are crucial for glucose homeostasis. Mammalian TAS2R, along with sensitizing molecules, is expressed in the intestinal mucosa, where it activates intestinal TAS2R to stimulate the secretion of glucagon-like peptide-1 (GLP-1) and other gut hormones by intestinal endocrine cells 107. Bitter melon extract (BME), whose bitter taste suggests TAS2R activation, has been extensively studied for its anti-diabetic properties. The constituents of bitter melon activate TAS2R, inducing the secretion of various hormones, notably GLP-1, by intestinal endocrine cells. GLP-1, a critical hormone, has emerged as a promising target for anti-diabetic drug development due to its role in regulating blood glucose levels through various mechanisms, including the modulation of insulin production. Upon TAS2R activation in intestinal endocrine cells, the β and γ subunits dissociate from the α subunit of the trimeric G proteins. This dissociation triggers the activation of phospholipase Cβ2 (PLCβ2), which catalyzes the production of diacylglycerol (DAG) and inositol 1,4,5-trisphosphate (IP3) from phosphatidylinositide at the cell membrane. IP3 binds to its receptor on the endoplasmic reticulum (ER) membrane, leading to Ca^2+^ release from the ER, thereby increasing the intracellular [Ca^2+^]. The subsequent increase in intracellular [Ca^2+^] leads to GLP-1 secretion by intestinal endocrine cells through mechanisms that remain to be fully elucidated 108. GLP-1 interacts with its receptor in the β and γ cells of the pancreas (GLP-1R) to stimulate the biosynthesis and release of insulin and somatostatin respectively, thereby contributing to the regulation of blood glucose levels 109. TAS2R activation initiates a cascade of intracellular events, including the dissociation of G protein subunits, activation of PLCβ2, production of DAG and IP3, and release of Ca^2+^ from the ER, culminating in GLP-1 secretion. GLP-1 then interacts with its receptor in pancreatic cells to regulate the release of insulin and somatostatin, contributing to the control of blood glucose levels. These findings underscore the potential of TAS2R agonists as therapeutic targets for T2DM.

#### 3.5.2 Obesity

Obesity, which affects approximately one-third of the global population, is increasingly recognized as a significant public health issue. Obesity is believed to be associated with altered gene expression in taste buds 110. As a sensory system, taste is the most intuitive indicator of our readiness to ingest food. Therefore, any alteration in this system can influence food intake and, consequently, body weight 111. The question of whether fat can be considered the "sixth taste" in humans has long been debated 112. New evidence supports the existence and function of TAS2R in various non-oral tissues. Specifically, TAS2R in gastrointestinal endocrine cells is involved in controlling appetite and regulating gut hormones that influence hunger and food intake 113. This indicates that region-selective targeting of TAS2R holds potential as a promising strategy for obesity treatment.

##### 3.5.2.1 Inhibition of ghrelin secretion

Gastrin, a potent appetite stimulant secreted by the stomach and consisting of 28 amino acids, targets the hypothalamus and brainstem. Gastrin signals hunger to the nervous system through ghrelin receptors, stimulating appetite and food intake while promoting the use of carbohydrates as a fuel source. Gastrin impedes fat utilization, contributing to weight gain; it also inhibits glucose-induced insulin secretion and stimulates gastrointestinal motility 114-117. In contrast, peptide YY (PYY), cholecystokinin (CCK), and GLP-1 influence a range of processes in the central nervous system, including the stimulation and inhibition of POMC/α-MSH neurons, gastrointestinal motility, and gastric emptying. These hormones induce a feeling of fullness by modulating food intake. Gastrin acts antagonistically to PYY, CCK, and GLP-1 signaling, amplifying appetite and enhancing food consumption by stimulating orexin activity, while suppressing the release of these gut-derived peptide hormones, ultimately contributing to obesity 118. Studies have demonstrated that subjects significantly reduce food intake after consuming a microencapsulated bitter ingredient (EBI) 119. Immunofluorescence studies reveal that gastrin co-localizes with taste complex G proteins. Bitter taste receptor agonists inhibit gastrin and motilin secretion via the taste G protein α-Gustducin, which in turn increases GLP-1, PYY, and CCK secretion, thereby reducing appetite 120. TAS2R agonists inhibit the secretion of gastrin and motilin, thereby increasing the secretion of GLP-1, PYY, and CCK, which in turn helps reduce appetite. This underscores the potential of targeting TAS2R as a therapeutic strategy for obesity and related metabolic disorders.

##### 3.5.2.2 Regulate gastrointestinal function

**Figure 7:**
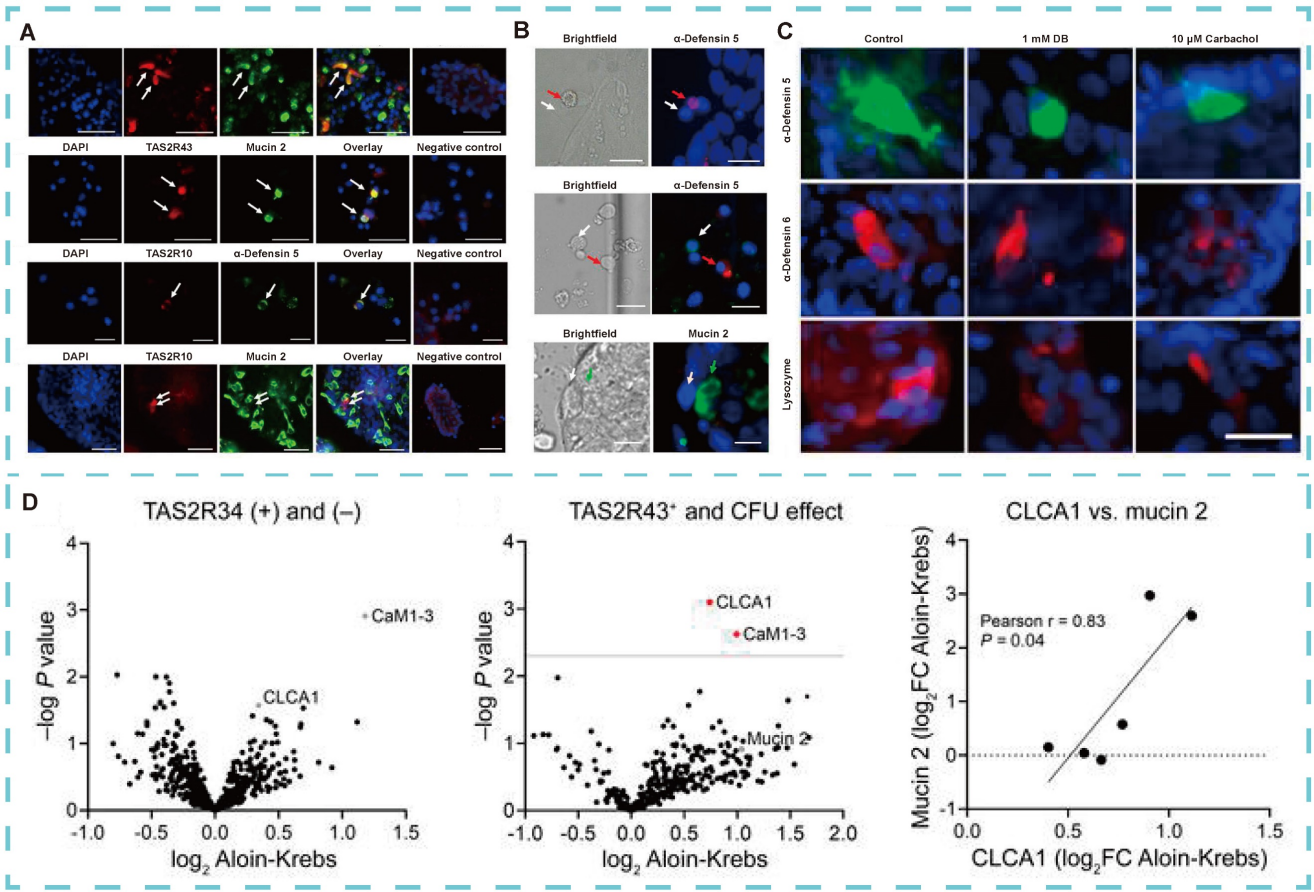
** Role of TAS2R in human intestinal physiology. (A)** Typical double fluorescence images of jejunal crypts in obese subjects. **(B)** Identification of immunostaining of cells in primary crypt foci of obese patients and the effect of bitter compound DB of intracellular Ca^2+^ changes. **(C)** Quantification of the effect of time-dependent protein expression in Paneth cells of lean and obese individuals. **(D)** Proteomic analysis of supernatant of jejunal crypts in obese patients after stimulation with bittering agents. Adapted with permission from 57, copyright 2021, American Society for Clinical Investigation.

Dysbiosis of the gut microbiota is recognized as a major contributor to obesity 121. Enteroendocrine cells (EECs), goblet cells, and Paneth cells regulate food intake and the secretion of hunger hormones in mice 34, 122. These cells are located in distinct regions of the gut, with Paneth cells residing at the base of the intestinal villi within the crypts. The crypts are tubular structures that form depressions in the intestinal wall, and their depth is inversely correlated with nutrient absorption capacity. Bitter compounds stimulate jejunal crypts, particularly in obese individuals, triggering the release of antimicrobial peptides. These peptides regulate obesity by protecting against pathogenic infections, preventing commensal bacterial antigen translocation, and modulating the gut microbiota 123, Notably, peptides such as alpha-defensin 5 and regenerating islet-derived protein 3 alpha (REG3A), along with innate immune factors like mucins and chemokines, are released in response to *E. coli* exposure. To confirm the physiological role of TAS2R in the human intestine, Kathrin I. Liszt's team 57 found that Paneth cells co-localize with TAS2R10 and TAS2R43, whereas goblet cells co-localize only with TAS2R43 in the jejunal crypts of obese individuals (**Figure 7A**). Stimulation of these crypts with bitter compounds induces the release of antimicrobial peptides and proteins (**Figure 7B-D**), thereby playing a role in obesity regulation.

Additionally, TAS2R expressed in gastrointestinal smooth muscle cells plays a critical role in regulating gut motility. Activation of TAS2R by bitter compounds enhances gastrointestinal motility, induces smooth muscle contraction, delays gastric emptying, and reduces inter-digestive gastric motility. These effects are mediated by Ca^2+^ elevation and ERK phosphorylation in human gastric smooth muscle cells (hGSMC), leading to earlier satiety and reduced nutrient intake. Furthermore, studies have identified pathways through which bitter compounds influence food intake by modulating the expression of GDF15, ADM2, and LDLR. The upregulation of these molecules following bitter compound ingestion triggers a conditioned taste aversion response to food stress, contributing to anorexic and weight-reducing effects. In summary, the activation of TAS2R in the gastrointestinal tract, which enhances gut motility and influences hunger signaling, presents a potential strategy for weight control 57.

##### 3.5.2.3 Reduce lipid accumulation

Kimura *et al.* 124 identified the expression of TAS2R108, TAS2R126, TAS2R135, TAS2R137, and TAS2R143 in murine adipose tissue, though the function of TAS2R in this tissue remains unclear. The team investigated the role of TAS2R in adipocytes through experiments analyzing gene expression changes in 3T3-L1 cells following bitter compound stimulation, along with TAS2R gene overexpression in preadipocytes. Their findings revealed that TAS2R gene expression was elevated in white adipose tissue (WAT) following exposure to bitter compounds or feeding in mice. An increase in TAS2R expression was also observed after the induction of 3T3-L1 adipocyte differentiation, bitter stimulation, and serum starvation. These findings suggest a potential role for TAS2R in 3T3-L1 adipocytes. Overexpression of TAS2R108 and TAS2R126 reduced the proliferation and differentiation of 3T3-L1 preadipocytes 125, whereas both overexpression and knockdown of TAS2R106 decreased lipid accumulation in adipocytes and reduced the expression of adipogenic genes. Although the specific functions of TAS2R remain to be fully elucidated, these findings suggest that TAS2R may play a significant role in regulating adipocyte function and lipid metabolism, thus offering a potential target for modulating adiposity and related metabolic processes.

### 3.6 The therapeutic potential of TAS2R as a target for preterm labor intervention

Current research suggests that TAS2R plays a crucial role in modulating physiological and pathological processes. The widespread expression of TAS2R across diverse tissues underscores their potential as promising molecular targets for drug delivery strategies. Therefore, exploring the expression and function of TAS2R is crucial for developing more effective and precise drug delivery systems. Preterm birth (PTB), defined as birth before 37 weeks of gestation, is a leading cause of infant illness and death worldwide. Each year, 15 million preterm births result in 1 million neonatal deaths. PTB affects 1 in 10 pregnancies in the U.S., impacting 500,000 newborns each year, and is associated with 70% of fetal health issues 126. Therefore, identifying innovative molecular targets that facilitate uterine relaxation, alongside the development of new classes of efficacious and safe tocolytics, is essential for advancing the management and treatment of PTB. Zheng *et al.* 127 demonstrated that activating bitter taste receptors exerts a potent relaxing effect on the contracted myometrium by inhibiting uterotonic-induced calcium (Ca²⁺) signaling pathways. Their studies revealed that this relaxation mechanism surpasses the efficacy of current tocolytic agents in preventing preterm births in two clinically relevant mouse models. Furthermore, activating the TAS2R signaling system in myometrial cells induces profound relaxation of the myometrium precontracted by a broad spectrum of contractile agonists. These findings suggest that targeting TAS2R is an ideal strategy for PTB treatment and highlight the potential for developing a novel class of tocolytics targeting the TAS2R family, offering a promising avenue for improved treatment and prevention of PTB. In summary, TAS2R is emerging as a key modulator of various physiological and pathological processes. These findings underscore the potential of TAS2R as a novel class of therapeutic targets, offering a promising avenue for developing more effective and precise drug delivery systems to address significant health challenges.

### 3.7 Role of TAS2R in cancer

#### 3.7.1 Head and neck squamous cell carcinoma

**Figure 8:**
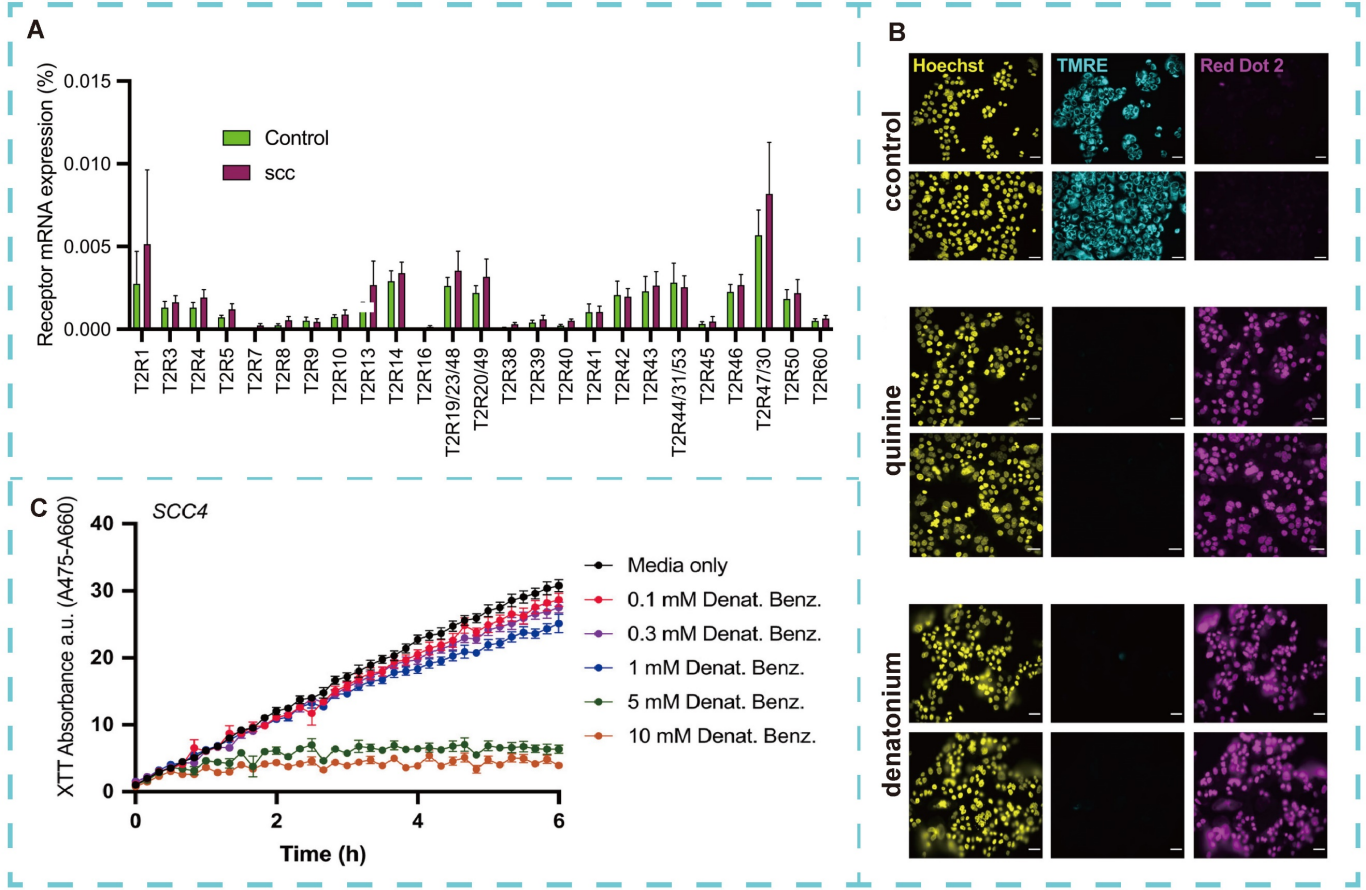
** Role of TAS2R in Head and Neck Squamous Cell Carcinoma. (A)** Quantitative reverse transcription PCR of TAS2R.** (B)** Bitter (T2R) agonists cause mitochondrial depolarization of HNSCC.** (C)** Bitter (T2R) agonists decrease cellular metabolism in HNSCC. Adapted with permission from 130, copyright 2021, John Wiley & Sons.

Head and neck squamous cell carcinoma (HNSCC) primarily affects regions such as the nasal cavity, sinuses, oral cavity, pharynx, and larynx 128. It is one of the most prevalent cancers, characterized by a high incidence and low survival rate. In addition to regional surgery, conventional cytotoxic chemotherapy, and radiotherapy, treatment options remain limited 129, highlighting the need for the exploration of targeted therapies. To determine whether TAS2R in HNSCC cells are functional, Carey *et al.* 130 evaluated various bitter compounds, assessing the responses elicited by agonists in calcium-loaded HNSCC cells. The findings demonstrated that bitter substances activate TAS2R-mediated Ca^2+^ influx, which triggers mitochondrial depolarization, caspase activation, and apoptosis. Buffering the nuclear Ca^2+^ elevation was shown to attenuate cysteine activation, indicating that TAS2R can induce apoptosis *in vitro* to inhibit HNSCC proliferation. Furthermore, an increase in the expression levels of TAS2R within HNSCC, as mapped within the cancer genome, was found to be significantly correlated with improved overall survival. The study suggests that TAS2R may serve as a therapeutic target to stimulate apoptosis and facilitate tumor-microbiome crosstalk in HNSCC, representing a potential biomarker for predicting outcomes and guiding therapeutic decisions (**Figure 8**).

#### 3.7.2 Pancreatic ductal adenocarcinoma

**Figure 9:**
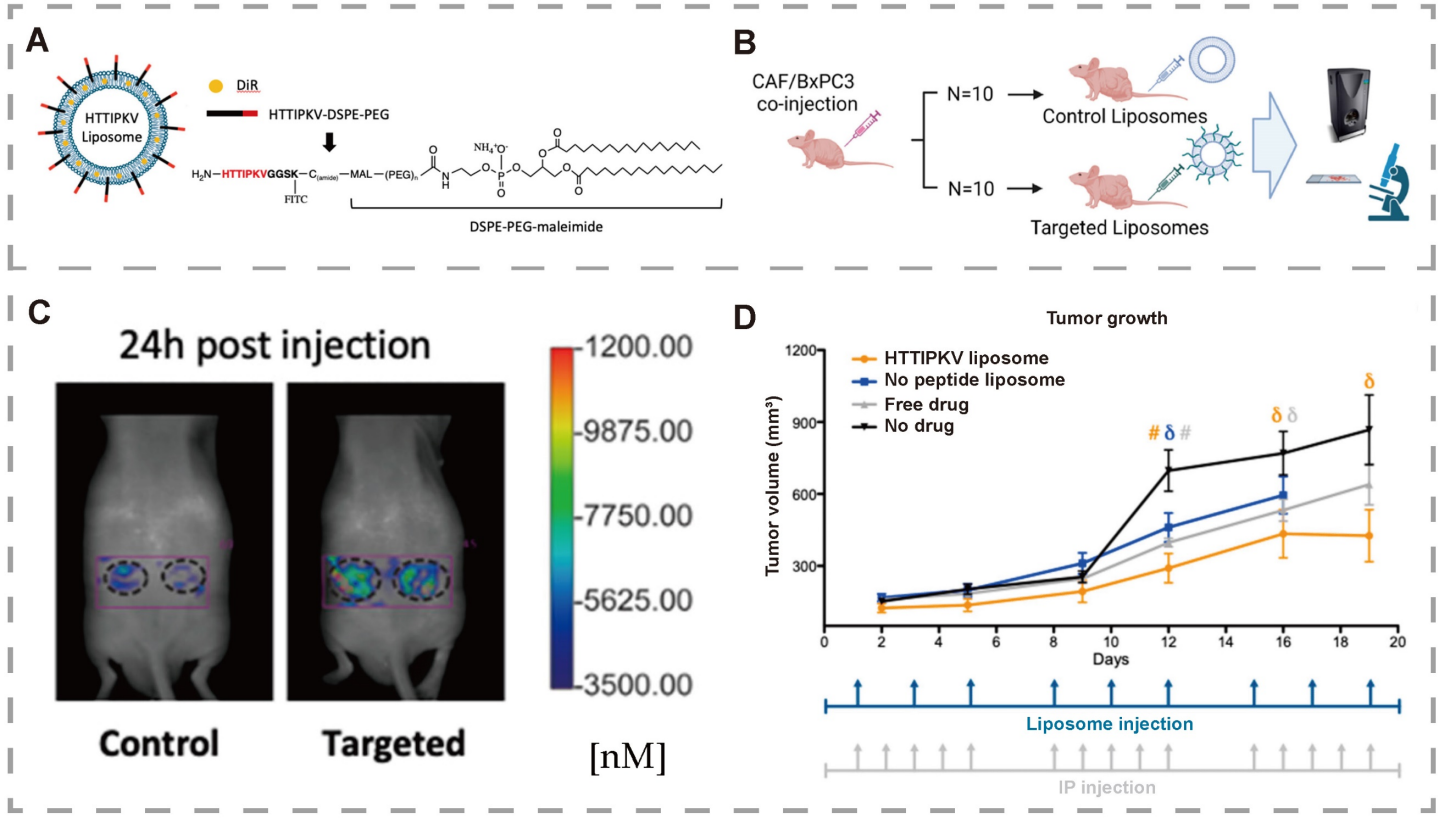
** TAS2R9 is a viable molecular target for Pancreatic Ductal Adenocarcinoma. (A)** Schematic of HTTIPKV liposome.** (B)** Schematic of mouse study design. **(C)** FMT images at 24 h post-liposome injection.** (D)** Average tumor volumes following different treatments in mice. Adapted with permission from 136, copyright 2023, MDPI.

Pancreatic ductal adenocarcinoma (PDAC), the most common subtype of pancreatic cancer (PC) 131, is a highly aggressive and lethal malignancy. PDAC arises when pancreatic cells acquire abnormal DNA mutations, enabling uncontrolled growth and division, which leads to tumor formation 132, 133. Due to the lack of characteristic early-stage symptoms and its aggressive nature, many PDAC patients are diagnosed at an advanced stage, when the cancer has already spread. Consequently, chemotherapy and radiotherapy remain the primary treatment options 134. PDAC exhibits multiple mechanisms of resistance to various drugs, which limit the effectiveness of these treatments. Understanding drug resistance at the molecular level is crucial for identifying novel therapeutic targets to restore drug efficacy and overcome chemotherapy resistance. This underscores the potential of bitter compounds and their receptors in addressing these challenges. Louisa Stern 135 was the first to report the expression and function of TAS2R10 in PDAC tissues and cell lines. Moreover, caffeine, a known ligand for TAS2R10, was found to sensitize tumor cells to the effects of two standard chemotherapeutic agents: gemcitabine and 5-fluorouracil. Knockdown of TAS2R10 in the BxPC-3 cell line diminished the efficacy of caffeine-induced sensitization. A plausible mechanism is that caffeine inhibits Akt phosphorylation by activating TAS2R10, leading to downregulation of ABCG2 expression. ABCG2 is a critical mediator of multidrug resistance, enabling cellular resilience against various chemotherapies. In summary, TAS2R10 functions in PDAC by downregulating chemoresistance in tumor cells, potentially improving patient outcomes. Additionally, Hung *et al.* 136 designed a TAS2R9-targeted liposome and demonstrated its ability to bind to TAS2R9 recombinant protein in a proof-of-concept drug delivery experiment. The study reports and validates TAS2R9 expression in pancreatic cancer-associated fibroblasts (CAFs), showing that TAS2R9 is a feasible target for therapy. The results show that TAS2R9 is a targeted receptor for HTTIPKV and a novel selective marker for CAFs (**Figure 9**). Higher liposome accumulation was observed in the targeted group compared to control liposomes, as shown using FMT system software. This indicated increased total drug exposure, and imaging via confocal microscopy revealed evident binding of liposomes to CAFs. In contrast to systemic administration, the average tumor volume in mice treated with targeted liposomes was significantly lower than in untreated mice. Collectively, these results show that the targeted liposome can effectively target tumor cells and restrain tumor development. These results suggest that TAS2R may play a significant role in drug delivery.

#### 3.7.3 Neuroblastoma

Neuroblastoma (NB), a malignancy originating from neural crest cells, is the most prevalent extracranial solid tumor in pediatric populations and can arise anywhere within the sympathetic nervous system 137. Unfortunately, current treatment regimens for high-risk neuroblastoma yield poor success rates, leaving survivors burdened with numerous long-term side effects from treatment 138. NB cells exhibit characteristics similar to cancer stem cells (CSCs), facilitating both tumorigenesis and metastasis. According to SEO 31, TAS2R8 and TAS2R10 are expressed in NB cells, where they play crucial roles in reducing stemness, migration, and invasion of these cancer cells. Similarly, ectopic transfection of these TAS2R in BE (2) C cells induced synaptic elongation, downregulated CSC markers (such as DLK1, CD133, Notch, and Sox2), and suppressed tumorigenicity. Moreover, overexpression of TAS2R suppresses cell migration, invasion, and matrix metalloproteinase activity. Notably, TAS2R also reduces the expression of hypoxia-inducible factor-1α, a pivotal regulator of tumor metastasis, along with its downstream targets, including vascular endothelial growth factor and glucose transporter-1 139. Collectively, these findings indicate that TAS2R can target CSCs by inhibiting cancer susceptibility traits and NB cell invasion, thereby highlighting their therapeutic potential in NB. This emphasizes the need for further investigation into the expression and function of TAS2R in NB and other diseases to facilitate the development of more effective and precise drug delivery systems.

#### 3.7.4 Acute myeloid leukemia

Acute myeloid leukemia (AML), a rare but highly aggressive hematological malignancy, is associated with a high mortality rate 140. Its pathogenesis is driven by epigenetic dysregulation, resulting from reciprocal translocations and mutations in transcriptional regulators and chromatin remodeling factors. These alterations lead to bone marrow differentiation arrest and an enhanced capacity for malignant self-renewal 141. In the complex interplay between AML cells and their microenvironment, membrane receptors initiate intracellular signals, enabling cells to respond to external stimuli and activate specific signaling pathways. Valentina *et al.* 142 characterized TAS2R expression in AML cells. Gene expression analysis revealed that activation of TAS2R by the prototypical agonist benzoic acid diamide significantly affected a range of genes involved in key AML processes. These molecular findings were supported by functional assays, which demonstrated that denatonium inhibited AML cell proliferation by inducing cell cycle arrest in the G0/G1 phase and triggering apoptosis through caspase cascade activation. Additionally, diammonium exposure reduced AML cell motility and migration, while simultaneously impairing cellular respiration by decreasing glucose uptake and oxidative phosphorylation. In conclusion, these findings extend previous observations of TAS2R expression in cancer cells to hematological malignancies, highlighting the role of TAS2R in the extrinsic regulation of leukemic cell functions. These emphasize the need for further investigation into the expression and function of TAS2R in AML and other diseases to develop more effective and precise drug delivery systems, offering a promising avenue for improving treatment and prevention of this devastating disease.

#### 3.7.5 Other cancers

TAS2R also plays significant roles in the pathophysiology and potential treatment of cancers such as breast, ovarian, and prostate cancer. Breast cancer remains a leading cause of cancer-related mortality among women, with approximately 26% of diagnoses annually and contributing to 14% of cancer-related deaths 10. Several therapeutic approaches are available for breast cancer treatment, including surgery, radiotherapy, chemotherapy, endocrine therapy, targeted therapy, and immunotherapy 143. However, these strategies often cause significant side effects, contribute to drug resistance, and demonstrate limited efficacy in certain cases, leading to suboptimal therapeutic outcomes. Singh *et al.* 10 reported reduced TAS2R4 expression and elevated TAS2R14 expression in breast cancer tissues. The activation of TAS2R4 and TAS2R14 was shown to suppress tumor cell migration and proliferation while promoting apoptosis through the MAPK/ERK1/2 and Gα12/13-RhoGTPase pathways, as well as GPCR transactivation. These findings suggest that TAS2R activation can inhibit tumor progression by modulating intracellular signaling pathways, with minimal adverse effects and a lower likelihood of drug resistance. Consequently, TAS2R represents a promising target for breast cancer therapy due to its multifaceted anti-tumor effects.

Prostate cancer ranks as the second most common malignancy among men and is the second leading cause of cancer-related deaths in this population 144. Patients with early-stage localized prostate cancer generally have a favorable prognosis, with a 10-year survival rate of 99%. However, metastatic prostate cancer drastically reduces the five-year survival rate to approximately 30%. High-grade serous ovarian carcinoma (HGSOC) is the most lethal gynecological malignancy worldwide, responsible for the majority of ovarian cancer-related deaths, with a five-year survival rate near 30% 145. These poor survival rates emphasize the need for novel therapeutic strategies for both prostate and ovarian cancer. Martin *et al.* 11 examined TAS2R expression in human epithelial ovarian and prostate cancer cells using qPCR analysis. Compared to the benign prostatic hyperplasia cell line (BPH1), most TAS2R genes were significantly downregulated in prostate cancer cells. Furthermore, noscapine treatment induced significant apoptosis in ovarian cancer cells. These findings suggest that TAS2R activation by bitter compounds can trigger apoptosis in tumor cells, highlighting TAS2R as a promising target for novel anticancer therapies.

## 4. TAS2R receptor agonists

Following the discovery of TAS2R, numerous bitter compounds have been screened for their interaction with cognate receptors, including drugs and food components from both natural and synthetic sources. Several bitter compounds have been identified as agonists for specific TAS2R isomers. Thousands of these compounds are well-documented and cataloged in existing databases. Among the diverse types of bitter compounds, polyphenols are particularly prominent 146. Based on this principle, we conducted a preliminary literature review to summarize the TAS2R targeted by various common bitter compounds and outline their physiological roles (**Table 2**).

## 5. Conclusion and outlook

Significant progress has been made in elucidating the expression of TAS2R beyond the oral cavity and their diverse physiological roles. These receptors extend beyond taste perception and toxic substance aversion, influencing multiple physiological functions. However, the mechanisms through which TAS2R modulate disease states, including osteomyelitis, cancer, and type 2 diabetes mellitus (T2DM), remain insufficiently explored. TAS2R can both suppress and exacerbate inflammation, underscoring the need for deeper insights into their dual roles in disease pathophysiology. While bitter taste perception is a well-established TAS2R function, their involvement in broader physiological processes remains poorly defined. The association between TAS2R polymorphisms and human lifespan presents a compelling avenue for further research. A major challenge is the scarcity of well-characterized TAS2R agonist formulations, as most available drugs exhibit mild bitterness, with limited investigation into their impact on patient adherence.

TAS2R regulate various physiological functions, including hormone secretion, gastrointestinal (GI) motility, and neural activation. Activation of these receptors stimulates neurohormone release, modulating food intake through peripheral effects on GI motility and central orexigenic signaling. These findings suggest that TAS2R could be targeted for obesity management, highlighting taste receptor modulation as a novel strategy for appetite control. TAS2R agonists have also been investigated for their signaling mechanisms and pharmacological effects, revealing expression in extraoral tissues such as myometrial and bone cells, as well as in cancers like pancreatic ductal adenocarcinoma (PDAC). This suggests their potential not only as therapeutic targets but also as biomarkers for disease progression. The characterization of numerous TAS2R agonists with favorable safety profiles in phase I clinical trials for metabolic and inflammatory diseases further supports their potential for broader therapeutic applications.

In summary, TAS2R exhibit diverse physiological roles extending well beyond taste perception. Emerging evidence suggests they should be considered not only as taste receptors but also as promising therapeutic targets for multiple diseases. This potential is particularly evident in respiratory diseases like asthma, where TAS2R activation has demonstrated airway relaxation and improved lung function. However, the full therapeutic potential of TAS2R remains to be elucidated. Further investigation is necessary to clarify TAS2R signaling mechanisms, receptor specificity, and potential side effects, along with their relevance in treating diseases beyond the respiratory system. Continued research is essential to fully unlock the therapeutic potential of TAS2R, paving the way for the development of targeted treatments across multiple conditions.

## Figures and Tables

**Table 1:** Distribution and expression of TAS2R in the normal human system

TAS2R	Localization in the human body	Method	Ref.
TAS2R1	Immune system, Cardio-vascular system, Excretory system, Nervous system, Endocrine system, Respiratory system, Skin	RT-PCR	17, 18
TAS2R3	Immune system, Endocrine system, Cardio-vascular system, Respiratory system, Reproductive system, Nervous system, Digestive system, Skin	ELCLIA	19, 20
TAS2R4	Immune system, Endocrine system, Cardio-vascular system, Respiratory system, Reproductive system, Nervous system, Digestive system, Excretory system Skin	IHC	21, 22
TAS2R5	Immune system, Endocrine system, Cardio-vascular system, Reproductive system, Excretory system, Nervous system, Digestive system, Skin	RT-PCR	23-25
TAS2R7	Immune system, Endocrine system, Cardio-vascular system, Reproductive system, Excretory system, Nervous system, Skin	IFC	26-28
TAS2R8	Immune system, Endocrine system, Cardio-vascular system, Excretory system, Nervous system, Skin	ChiP	29-31
TAS2R9	Immune system, Endocrine system, Cardio-vascular system, Excretory system, Nervous system, Skin		32
TAS2R10	Immune system, Endocrine system, Cardio-vascular system, Reproductive system, Excretory system, Nervous system, Digestive system, Skin	WB	31, 33-36
TAS2R13	Immune system, Endocrine system, Cardio-vascular system, Reproductive system, Excretory system, Nervous system, Skin	IFC	37
TAS2R14	Immune system, Respiratory system, Cardio-vascular system, Endocrine system, Reproductive system, Excretory system, Nervous system, Skin	RT-qPCR	38-40
TAS2R16	Respiratory system, Endocrine system, Excretory system, Cardio-vascular system, Nervous system, Skin	IFC	41, 42
TAS2R19	Immune system, Reproductive system, Cardio-vascular system, Excretory system, Skin	RT-PCR	43, 44
TAS2R20	Immune system, Endocrine system, Cardio-vascular system, Reproductive system, Excretory system, Nervous system, Digestive system, Skin	RT-qPCR	45
TAS2R30	Immune system, Endocrine system, Cardio-vascular system, Reproductive system, Excretory system, Digestive system, Skin	RT-qPCR	46, 47
TAS2R31	Immune system, Endocrine system, Cardio-vascular system, Excretory system, Skin	RT-qPCR	48, 49
TAS2R38	Respiratory system, Immune system, Endocrine system, Cardio-vascular system, Excretory system, Nervous system, Adipose tissue, Digestive system, Skin	RT-PCR	50-53
TAS2R39	Respiratory system, Immune system, Endocrine system, Cardio-vascular system, Reproductive system, Excretory system, Nervous system, Skin	RT-PCR	45, 54
TAS2R40	Immune system, Endocrine system, Cardio-vascular system, Excretory system, Nervous system, Skin	RT-qPCR	49, 55
TAS2R41	Immune system, Cardio-vascular system, Endocrine system, Excretory system, Nervous system, Skin	RT-qPCR	56
TAS2R42	Respiratory system, Immune system, Endocrine system, Reproductive system, Cardio-vascular system, Excretory system, Nervous system, Skin	PCR	29, 46
TAS2R43	Respiratory system, Cardio-vascular system, Immune system, Endocrine system, Reproductive system, Nervous system, Excretory system, Digestive system, Skin	RT-PCR, WB, IFC	57, 58
TAS2R44	Nervous system	RT-PCR	59
TAS2R45	Respiratory system, Immune system, Endocrine system, Cardio-vascular system, Reproductive system, Nervous system, Excretory system, Skin	RT-qPCR	60
TAS2R46	Respiratory system, Immune system, Endocrine system, Cardio-vascular system, Nervous system, Skin	RT-qPCR	61
TAS2R47	Respiratory system	RT-PCR	62, 63
TAS2R49	Immune System, Endocrine System, Cardiovascular System, Reproductive System, Excretory System, Nervous System, Digestive System, Skin, Respiratory System	RT-qPCR	64
TAS2R50	Respiratory system, Immune system, Endocrine system, Cardio-vascular system, Reproductive system, Nervous system, Skin	RT-qPCR	64
TAS2R60	Immune system, Cardio-vascular system, Nervous system, Skin	qPCR	65

**Table 2:** Common bitter receptor agonists

Compound	TAS2R	Main function	Ref
Chinese gentian	TAS2R1	Inhibit fat production and reduce daily energy intake	119, 147
Quinine	TAS2R4, 7,10,14, 31,39,40,43,46	Regulate lipogenesis and treat chronic sinusitis	148, 149
Chloroquine	TAS2R3	Relieve respiratory discomfort	150
1,10-phenanthroline	TAS2R5	Causes relaxation of airway smooth muscle	23
Diammonium benzoate	TAS2R8, 13,47	Regulate metabolism	151
Caffeine	TAS2R10,14,43,46	Induce gastric parietal cells to secrete gastric acid and down-regulate drug resistance of PDAC cells	135, 152
Epicatechin	TAS2R14, 39	Enhance satiety and reduce food intake.	153
Saccharin	TAS2R31, 43	Can dilate bronchi.	154
Quercetin	TAS2R14	Play a neuroprotective role.	155
Naringenin	TAS2R4, 14	Bronchiectasis, anti-inflammatory, anti-tumor	156-158
Andrographolide	TAS2R50	Antioxidant and anti-inflammatory.	159
Diphenhydramine	TAS2R14	Central nervous depressant	91
Vanillin	TAS2R14, 20,39	Enhance appetite and improve the composition of the gastrointestinal microbiota.	45
Piperine	TAS2R14	Stimulation of glucagon-like peptide-1 secretion.	33
